# Clinical and radiological predictors of response to lumbar transforaminal epidural steroid injection at 3 months: A retrospective study

**DOI:** 10.1016/j.inpm.2022.100159

**Published:** 2022-11-15

**Authors:** Anuj Jain, Suruchi Jain, Swapnil Kumar Barasker, Saurabh Trivedi, Ekta Gupta, Ashutosh Kaushal

**Affiliations:** a Department of Anesthesiology, All India Institute of Medical Science, Saket Nagar, Bhopal, Madhya Pradesh, 462020, India; b Department of Nuclear Medicine, All India Institute of Medical Sciences, Bhopal, Madhya Pradesh, 462020, India; c Department of Anesthesiology, Sri Aurobindo Medical College and Post Graduate Institute, Indore, Madhya Pradesh, 453111, India; d Department of Anesthesiology, Chirayu Medical College and Hospital, Bhopal, Madhya Pradesh, 462020, India

**Keywords:** Intervertebral disc displacement, Low back pain, Leg pain, Epidural steroid injection, Magnetic resonance imaging, Predictors

## Abstract

**Background:**

Transforaminal epidural steroid injection (TFESI)^1^ is commonly used to relieve pain due to intervertebral disc displacements (IDD).^2^ Poorly defined selection criteria lead to post procedure dissatisfaction among patients. We aimed to find clinical and magnetic resonance imaging (MRI)^3^ predictors for pain relief at 3 months following a TFESI. Poorly defined selection criteria lead to post procedure dissatisfaction among patients.

**Methods:**

A retrospective study of 116 patients who had undergone TFESI. Predictors used were - age, duration of symptoms, body mass index, neuropathic character of pain, dermatomal distribution of pain, claudication distance, response to anti-neuropathic medication and extent of nerve root compromise in MRI as per Pfirmann criteria. A relief of 50% or more at the end of 3 months was considered the criterion for significant pain relief from TFESI.

**Results:**

At 3 months, 72% (84/116) had significant pain relief. Dermatomal distribution of pain (73%) and neurogenic claudication (71%) were the most prevalent clinical features. Dermatomal distribution of leg pain, responsiveness to anti-neuropathic medications and a Pfirmann grade 2/3 in MRI were the most important predictors with an odds ratio (OR)^4^ of 12.1, *P* ​< ​0.001, OR 6.4, *P* ​= ​0.002 and OR 3.1, *P* ​= ​0.056, respectively. The model was statistically significant χ^2^ (3, N ​= ​116) ​= ​43.43,*P ​<* 0.001 and explained 52% variance in the outcome. The model correctly predicted the outcome 85% times.

**Conclusions:**

If a patient has leg pain which is dermatomal in distribution, responds to anti-neuropathic medications and has Pfirmann grade 2/3 in MRI, then chances of more than 50% relief persisting at 3 months after TFESI are significantly better.

## Abbreviations

TFESI: Transforaminal Epidural Steroid Injection
IDD: Intervertebral disc displacements
MRI: Magnetic resonance imaging
NRS: Numerical rating scale
BMI: Body mass index
OR: Odd's ratio

## 1 Introduction

A lumbar transforaminal epidural steroid injection (TFESI) is option to palliate the agony associated with a lumbar intervertebral disc displacement (IDD). However, the criteria for selecting a patient for an TFESI are subjective and vary among physicians. There is considerable variability in the choice of intervention for delivering the local anaesthetic and steroids to the desired lumbar IDD. Newer agents and techniques have been employed, but TFESI is still the most commonly used method [1,2].

Various investigators have attempted to identify factors that can predict the outcome of an TFESI; most of the available studies focus on observations during or after placement of TFESI to predict the efficacy in future follow-ups [3,4]. In this retrospective study, we have attempted to identify clinical and MRI features that can be used to predict the result of an TFESI in relieving lumbar IDD associated leg pain. Since these predictors are routinely available to a physician before the procedure, the results of this study can help the physicians to choose their patients more wisely.

## 2 Materials and methods

**Fig. 1:**
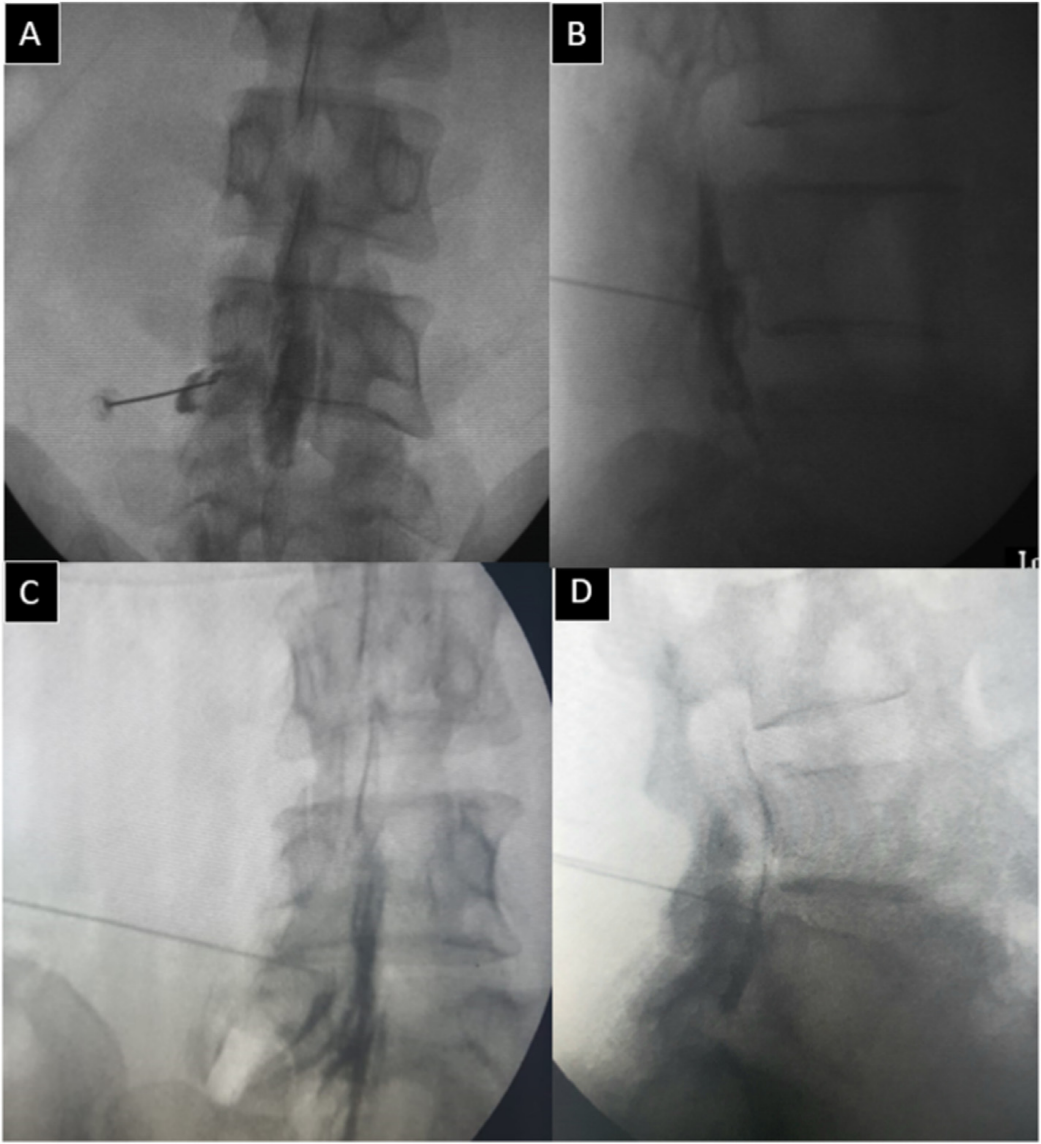
Fluoroscopic images of TFESI in the sub-pedicular approach and lateral recess approach. (A) Anteroposterior view showing the acute angle of the needle and (B) lateral view with needle tip in superior part of foramen in sub-pedicular approach. (C) We see the much flatter needle trajectory in anteroposterior view of lateral recess approach and (D) lateral view showing needle tip in the lower part of the foramen.

In this retrospective study, records of patients of low back pain (LBP)^5^ and unilateral radicular leg pain were analyzed. Exemption was obtained from the institutional review board (IHECLOP/2019/IM0237). Choice of TFESI depended on the location of IDD; for paracentral IDD, TFESI was given in the lateral recess [5]; foraminal and extraforaminal disc displacements were treated by the sub-pedicular approach of TFESI. (Fig. 1). Intervertebral level for the procedure was decided based on nerve root compromise in MRI. If the nerve root compromise existed at more than one adjacent level, then TFESI was given sequentially on both the levels. The clinically significant level was given preference.

For the assessment of pain severity and follow ups, a 11-point Numerical rating scale (NRS)^6^ was used. A TFESI was considered correctly placed if the pain was relieved by 80% or more immediately after the procedure; 50% or more pain relief at 3 months after TFESI, was considered clinically significant. Patients report of NRS before, and after TFESI were used to derive the percentage improvement.

Patients not giving consent for TFESI or having with a neurological deficit, uncontrolled diabetes mellitus, or local site skin infections were not given TFESI due to our clinical protocol. Records of any patient who received a spine steroid injection with in the past 3 months, connective tissue disorder, peripheral vascular disease, spondylolisthesis, advanced spondyloarthropathy were also excluded. Patients with incomplete follow-up data were also excluded.

Duration of symptoms, body mass index (BMI),^7^ character of pain (neuropathic/non-neuropathic) based on DN4 questionnaire (score 4 or more), distribution of pain (dermatomal/non-dermatomal), claudication distance (for the purpose of classification we used an arbitrary distance of 250 ​m, claudication was considered absent if the patient could walk more than 250 ​m) extent of nerve root compromise in MRI (based on Pfirmann classification) [6], and analgesic response to anti-neuropathic drugs (pregabalin, gabapentin, carbamazepine and duloxetine, alone or in combination were the most commonly used molecules in our clinical practice) were included in the analysis. The patient's distribution of leg pain was used determine the pattern and to clinically assess the level of nerve root compromise. While dealing with patients with leg pain we often come across patients who cannot exactly define the distribution of pain, they say that a particular region aches, that painful region may span multiple dermatomes. If the pain in leg spanned multiple dermatomes rather than a particular dermatome, we considered it to be nondermatomal; if the distribution of patient's pain was along a particular anatomical dermatome [7], it was classified as dermatomal pain.

Neurodynamic tests (straight leg raise test, crossed straight leg raise test, and femoral stretch test) were not included as predictor due to poor diagnostic performance, specifically in chronic low back pain [8].

Nerve root effacement with obliteration of perineural fat or nerve root compression with loss of perineural fat were considered essential to consider any nerve root compromise as significant, which is consistent with Pfirmann grade 2 or 3 [6,9].

If the patients opined that there was no reduction in pain or an equivocal response, and those who could not continue anti-neuropathic medication due to side effects, they were labeled non-responders.

Sample size calculation was done based on the method suggested by Green et al. [10], and a sample size of 113 was found to be adequate.

### 2.1 Statistical analysis

Statistical analysis was done using SPSS-16 software. Correlation between the outcome at 3month and the binary predictors was studied using the Chi-squared test. The predictors that had a statistically significant correlation with the outcome were checked for collinearity and then were used in the Binary logistic regression analysis to generate a potential predictor model. In logistic regression we used the ‘Enter’ method. A *P*-value of 0.05 or less was considered as statistically significant.

## 3 Results

**Fig. 2:**
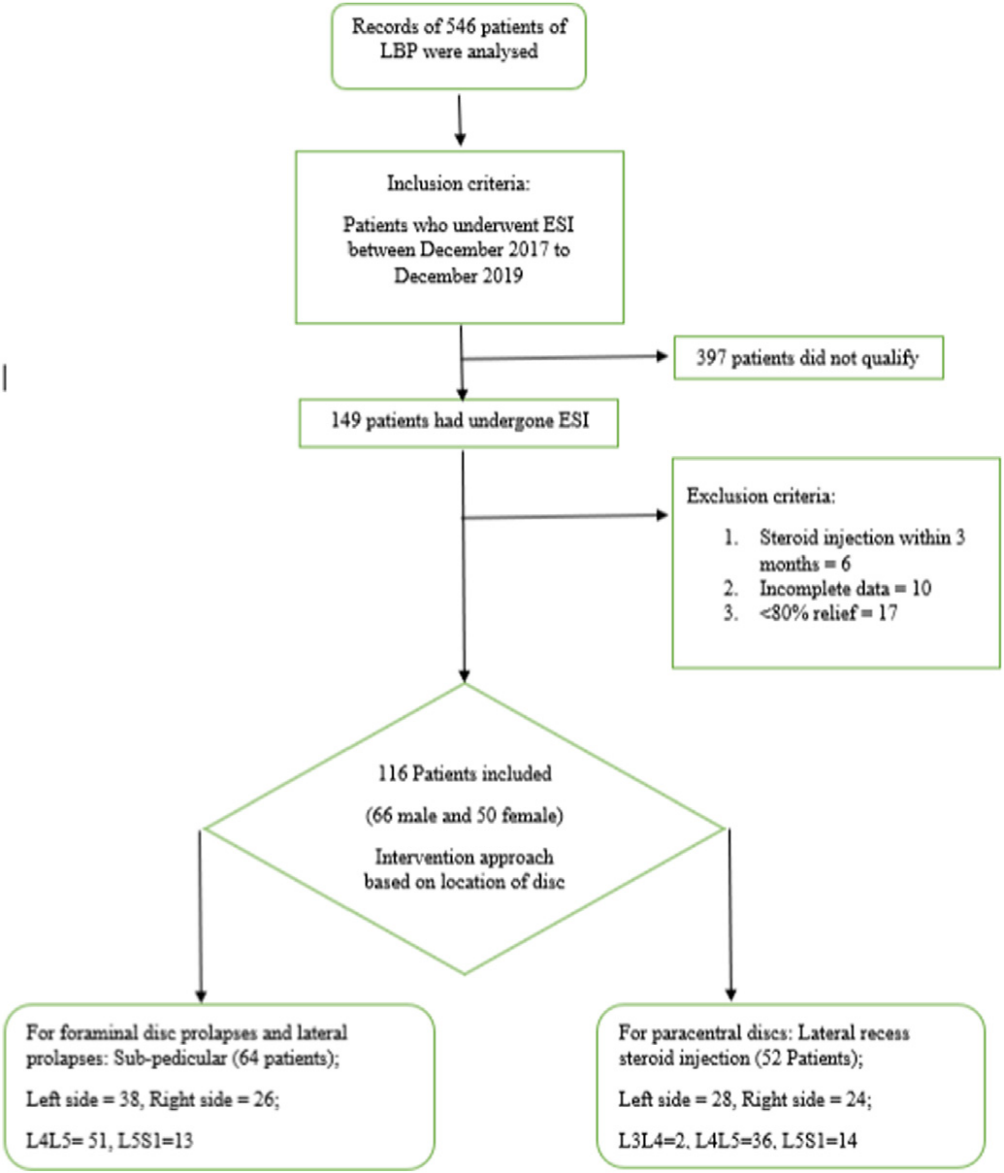
Study summary LBP; Low back pain, TFESI; Transforaminal Epidural steroid injection.

We analyzed the records of 546 patients who had undergone treatment for LBP at a tertiary care teaching hospital in central India between July 2017 and July 2019. Out of the 546 patients, 149 patients had undergone a TFESI. Records of six patients who had undergone steroid injection within the previous 3 months were excluded. Ten patients were excluded as their data was incomplete. In 17 patients the immediate relief after TFESI was less than 80%, which was considered a technical failure in this study, and such patients were excluded. 116 patients who had completed a follow-up of 3 months were included in the analysis; among them 66 were male and 50 were female. Most commonly encountered intervertebral level was L4-5 (86/116) followed by L5S1 (28/116) and L3-4 (2/116). (Fig. 2).

The demographic data is presented in Table 1. At the end of 3 months, 72% had significant pain relief. Sub-pedicular TFESI was given in 55% (64/116) patients while the rest received TFESI in lateral recess. 67% and 77% patients in the sub-pedicular and lateral recess steroid injection group had pain relief at the end of 3 months, *P* ​= ​0.270.

**Table 1:** Demographic data.

S.no.	Variable	
1.	Age (Mean ​± ​SD)	42.1 ​± ​1.8 years
2.	Gender (%)	Male ​= ​56.90% (66/116) Female ​= ​43.10% (50/116)
3.	BMI (Mean ​± ​SD)	26.0 ​± ​1.87 ​kg/m^2^
4.	Duration of symptoms (Mean ​± ​SD)	4.5 ​± ​1.3 months

Prevalence of each predictor under study is tabulated in Table 2.

**Table 2:** Prevalence of important clinical predictors in the study cohort.

S.no.	Predictors	Prevalence (%)
1	Dermatomal distribution of leg pain.	73
2	BMI (25 or more)	67
3	Neurogenic claudication	71
4	DN4Q score ≥4	60
5	Anti-neuropathic drug response	69.7
6	Pfirmann of grade ≥2	54

DN4Q: Douleur neuropathique 4 5.

Among all the parameters under study, a dermatomal distribution of pain, responsiveness to anti-neuropathic medications and a Pfirmann grade 2 or more on MRI had a significant correlation (Table 3).

**Table 3:** Summary of the outcome predictors at 3months time point.

Predictor (N ​= ​116)		Successful outcome at 3months	Failure outcome at 3months	P-value
Dermatomal distribution	+	71 (61.21%)	15 (12.93%)	<0.001∗
	–	10 (8.62%)	20 (17.24%)	
BMI>25	+	63 (54.31%)	21 (18.10%)	0.07
	–	18 (15.52%)	14 (12.07%)	
Gender	Male	42 (36.21%)	21 (18.10%)	0.41
	Female	39 (33.62%)	14 (12.07%)	
Claudication distance ≤250 ​m	+	63 (54.31%)	21 (18.10%)	0.05
	–	18 (15.52%)	14 (12.07%)	
DN4Q score≥4	+	54 (46.55%)	19 (16.38%)	0.2
	–	27 (23.28%)	16 (13.79%)	
Anti-neuropathic drug response	+	67 (57.76%)	15 (12.93%)	<0.001∗
	–	14 (12.07%)	20 (17.24%)	
Pfirmann of grade ≥2	+	53 (45.69%)	14 (12.07%)	0.01∗
	–	28 (24.14%)	21 (18.10%)	

∗Indicates a P-value <0.05 is significant. BMI: Body mass index, DN4Q: Douleur neuropathique 4 question.

Binary logistic regression analysis was performed and only factors that had a significant correlation were included (Table 4, Table 5). The model was statistically significant, χ^2^ (3,N ​= ​116) ​= ​43.43, *P ​<* 0.001; the model explained between 35.5% (Cox and Snell R square) and 52% (Nagelkarke R^2^) of the variance in the dependent variable and correctly classified 85% of the cases. Result of the Hosmer and Lemeshow goodness of fit test was not significant χ2 (5,N ​= ​116) ​= ​5.07,*P ​=* 0.41 and it showed that the model correctly predicted a successful outcome 97.5% times (8.75/9).

**Table 4:** Summary of the linear regression analysis. Independent variables that had significant correlation with the outcome were included in the analysis.

	B coefficient	SE	df	Exp(B)	95% CI of Exp(B)	P-value
Dermatomal distribution	2.49	0.62	1	12.1	3.56–41.1	<0.001∗
Anti-neuropathic drug response	1.86	0.60	1	6.4	1.98–20.97	0.002∗
Pfirmann grade ≥2	1.14	0.59	1	3.1	0.97–10.16	0.056
Constant	−2.42	0.70	1	0.08		0.001∗

∗indicates a p-value <0.05 is significant.

**Table 5:** Correlation matrix showing the correlation between the various predictors.

	Constant	Dermatomal distribution	Anti-neuropathic drug response	Pfirmann grade ≥2
**Constant**	**1.0**	**−0.67**	**−0.56**	**−0.45**
**Dermatomal distribution**	**−0.67**	**1.0**	**0.12**	**0.14**
**Anti-neuropathic drug response**	**−0.56**	**0.12**	**1.0**	**−0.12**
**Pfirmann grade** ≥2	**−0.45**	**0.14**	**−0.12**	**1.0**

## 4 Discussion

In this study we found that in patients with IDD leg pain having a dermatomal distribution, positive response to anti-neuropathic medication and having Pfirmann grade 2 or more are more likely to have a more than 50% relief in leg pain at the end of 3 months following a TFESI. Among the three predictors, dermatomal distribution of leg pain appears to be the most important predictor followed by a positive response to anti-neuropathic drugs. MRI findings did not significantly contribute the predictor model.

Lumbar IDD associated leg pain may be due mechanical and/or chemical irritation of the nerve. The site of this nerve root compromise lies in the ventral epidural space. IDD is a dynamic process and can either improve or worsen with time [11]. Clinical presentation of acute and chronic pain in lumbar IDD may be different. In acute phase the pain is more well defined. As the pain becomes chronic windup phenomenon and the recruitment of wide dynamic range of neurons tend to make it more ill-defined [12,13].

In this study we found that leg pain is likely to respond better if the dermatomal nature of the pain is still preserved. Leg pain is not specific of lumbar IDD and may arise due to problems of the musculoskeletal system. Leg pain of IDD origin is more likely to manifest as neuropathic pain, but a significant proportion of the patients may not have classical features of neuropathic pain. In this study we found that a DN4Q score of 4 or more was not a good predictor of response to TFESI. Besides, this study also highlights that if the leg pain is of neuropathic origin, it is likely to respond to anti-neuropathic drugs and this response to anti-neuropathic drugs may act as an important pre-procedure predictor of a successful outcome.

Bogduk et al. [4], reported that the success of TFESI does not have a significant association with size, site or size of herniation as seen in MRI. In our study we found that nerve root compromise of Pfirmann grade 2 or more had a significant correlation with the 3month outcome, but it failed to have a significant impact in the predictor model. Ekedahl et al. [14], also observed that a high-grade nerve root compromise was predictive of successful outcome of ESI.

Our study found that BMI and duration of pain are not good predictors of relief 3 months after TFESI. Logically chances of central sensitization and unwinding should increase with time and hence the results should have worsened with increasing duration of pain; the results of our study highlight that natural course of the IDD progression is multifactorial and duration of the illness is one but not the only factor. Ghahreman A et al. [4],also found duration of symptoms is not a good predictor for outcome. Cyteval et al. [15], found that duration of symptoms was highly predictive of TFESI outcome at 2 weeks; in their study average duration of symptoms was 3 months in the excellent pain relief (75% or more) group, while 8 months in the poor relief group (25% or less). In our study, a shorter duration of symptoms, less stringent criteria to consider the procedure successful and an injection technique customized to the site of disc herniation can be possible reasons for the difference observed.

This study also found that claudication distance does not have significant correlation with the outcome at 3 months follow-up, although the results were borderline. Our results are in sync with findings of McCormick et al. [16], who identified that absence of neurogenic claudication was an important predictor of satisfactory pain relief.

### 4.1 Limitations

The current study has multiple limitations, the most important being the retrospective design of the study. The patient data of only small number of patients could be analyzed, a prospective study with larger sample size would help in ascertaining the results. Functional improvement was not studied and only relief in pain was assessed. Addition of electrodiagnostic studies to the above criteria can further help improve the prediction of post-procedure results [17]. The high clinical success rate of the study may prevent the full understanding of the predictive power of many of the studied factors. We purposely did not evaluate intra and post procedure factors in the study, as the main aim was to determine pre-procedure factors.

## 5 Conclusion

The findings of this study support that preprocedural characteristics of dermatomal leg pain, response to neuropathic pain medications, and Pfirrman score of 2 or 3 on MRI demonstrating nerve compression are associated with greater than 50% pain relief at 3 months after TFESI among patients with unilateral radicular pain.

## Funding

Nil.

## Declaration of competing interest

The authors declare that they have no known competing financial interests or personal relationships that could have appeared to influence the work reported in this paper.

## Footnotes

5 Low back pain.

6 Numerical rating scale.

7 body mass index.
